# Prediction of Histologic Subtype and FNCLCC Grade by SUVmax Measured on ^18^F-FDG PET/CT in Patients with Retroperitoneal Liposarcoma

**DOI:** 10.1155/2021/7191363

**Published:** 2021-01-06

**Authors:** Cheng-Peng Li, Dao-Ning Liu, Ni-Na Zhou, Xiu-Yun Tian, Zhen Wang, Bo-Nan Liu, Chun-Yi Hao

**Affiliations:** ^1^Key Laboratory of Carcinogenesis and Translational Research (Ministry of Education), Sarcoma Center, Peking University Cancer Hospital and Institute, No. 52 Fu-Cheng Road, Hai-Dian District, Beijing 100142, China; ^2^Key Laboratory of Carcinogenesis and Translational Research (Ministry of Education), Department of Nuclear Medicine, Peking University Cancer Hospital and Institute, No. 52 Fu-Cheng Road, Hai-Dian District, Beijing 100142, China

## Abstract

This study aimed to evaluate the usefulness of maximum standardized uptake value (SUVmax) on ^18^F-fluorodeoxyglucose positron emission tomography with computed tomography (^18^F-FDG PET/CT) in differentiating the subtypes and tumor grades of retroperitoneal liposarcoma (RPLS). The data of RPLS patients who underwent surgical resection from November 2013 to December 2019 at the sarcoma center of our institute were reviewed. The demographics, clinical features, and SUVmax of 84 patients who underwent preoperative ^18^F-FDG PET/CT scans were analyzed. Of these, 19 patients (22.6%) were with well-differentiated liposarcoma (WDLPS), 60 patients (71.4%) were with dedifferentiated liposarcoma (DDLPS), and 5 patients (6.0%) were with pleomorphic liposarcoma (PMLPS). The median SUVmax of WDLPS, DDLPS, and PMLPS groups was 2.8 (IQR: 1.9–3.2), 6.2 (IQR: 4.1–11.3), and 4.5 (IQR: 4.0–7.4). The ROC curve suggested 3.8 as an approximate cutoff value of SUVmax for distinguishing WDLPS and non-WDLPS (sensitivity = 0.769; specificity = 0.895). The median SUVmax for FNCLCC Grades 1, 2, and 3 of RPLS was 2.5 (IQR: 1.9–3.2), 4.5 (IQR: 3.2–6.7), and 9.0 (IQR: 6.0–13.3). The ROC curves suggest that SUVmax of ≤3.8 and >5.3 can be used for predicting FNCLCC Grades 1 and 3, respectively. The result showed that ^18^F-FDG PET/CT exhibited high sensitivity and specificity for identifying the subtypes and FNCLCC grades of RPLS. Additionally, ^18^F-FDG PET/CT might be a useful complementary imaging modality for guiding suitable biopsy location of RPLS.

## 1. Introduction

Retroperitoneal sarcomas (RPSs) are rare tumors, with an expected incidence of 3 new cases per 1,000,000 people each year in mainland China [1]. Surgical resection is the mainstay of curative therapy for treating RPS. Previous studies have proved that histologic subtype and tumor grade of RPS are considered as significant predictors of survival [2, 3]. Liposarcoma is the most common type of sarcomas that arises in the retroperitoneum. However, the difficulties in resection of tumors with wide clear margins and predilection for postoperative local recurrence contributed to the complexity of surgical treatment of retroperitoneal liposarcoma (RPLS).

Anatomic imaging including computed tomography (CT) and magnetic resonance imaging (MRI) are widely employed in the diagnosis, staging, and follow-up of RPLS, but both these were associated with limited capabilities in evaluating biological activity and malignant capacity of tumors. The ^18^F-fluorodeoxyglucose positron emission tomography with computed tomography (^18^F-FDG PET/CT) combines anatomic localization by CT with functional PET imaging and has a promising role in the diagnosis and treatment of many solid tumors. It is associated with high sensitivity, specificity, and safety in evaluating glucose metabolism of tumors for staging and restaging. The maximum standardized uptake value (SUVmax) refers to the radioactivity concentration of imaging agent in the tumor, which is the most widely used semiquantitative parameter. However, there are only a few studies on the use of ^18^F-FDG PET/CT in RPLS due to its rarity and low incidence [4–6].

Therefore, a monoinstitutional series of RPLS in differentiating the subtypes and tumor grades by SUVmax by using ^18^F-FDG PET/CT was conducted.

## 2. Materials and Methods

### 2.1. Study Design

This study was conducted according to the ethical guidelines of the 1975 Declaration of Helsinki. All patients provided written informed consent form, and the study protocol was approved by the Institutional Ethics Committee of the Peking University Cancer Hospital (approval no. 2019KT19). A retrospective review of our institutional RPS database and patients' clinical charts was conducted.

The following formula for estimating the sample size of this study was used:

(1) $$\begin{array}{c} n=\frac{Z_{\alpha /2}^{2}P\left(1-P\right)}{d^{2}}, \end{array}$$

where *d* = 0.1 and for *α* = 0.05, *Z*_*α*/2_ is inserted by 1.96. The sample size was calculated to be 47 or 65 when the value of *P* is defined as sensitivity (85.7%) or specificity (78.3%) as reported in the previous study [6]. Thus, the study requires enrollment of at least 65 patients for obtaining adequate sensitivity/specificity.

The inclusion and exclusion criteria were as follows: (1) patients who underwent operations with curative intent of resection and whose postoperative pathological diagnoses were RPLS were included; (2) patients who underwent excisional biopsies by open and laparoscopic procedures were excluded as the biopsied specimen might not reflect the histology of RPLS due to its heterogeneity; (3) patients with RPS other than liposarcoma were excluded; (4) patients with primary limb/trunk liposarcoma metastasizing to retroperitoneum were excluded; and (5) patients who received any antitumor treatment including chemotherapy, radiotherapy, targeted therapy, or immunotherapy before undergoing ^18^F-FDG-PET/CT scan were excluded because of their possible impact on tumor metabolism.

Clinicopathological features including preoperative SUVmax as measured by ^18^F-FDG PET/CT scan and pathological data (tumor size, grade, and histology) were collected. The histology of all patients was independently reviewed by two experienced pathologists with special expertise in sarcomas. In case of any disagreement between the two pathologists, a third pathologist was consulted for reaching a consensus regarding the diagnosis. According to the WHO classification of soft tissue tumors, histologic types were grouped as follows: well-differentiated liposarcoma (WDLPS), dedifferentiated liposarcoma (DDLPS), myxoid liposarcoma (MLPS), and pleomorphic liposarcoma (PMLPS). The tumor was graded by the French Federation of Cancer Centers Sarcoma Group (Fédération Nationale de Centres de Lutte Contre le Cancer (FNCLCC)) system [7].

### 2.2. PET/CT Examinations

Patients were instructed to fast for at least 6 hours prior to undergoing PET/CT scan. The images were acquired approximately 60 minutes after an injection of 3.7 MBq/kg ^18^F-FDG. A whole-body acquisition was commenced in 6−8 bed positions (1 min/bed) using a hybrid system (Gemini TF 16 PET/CT, Philips, Netherlands), covering from the base of the skull to the upper thigh. CT was conducted using the following parameters: 120 kV, 100 mAs, and slice thickness of 3 mm for attenuation correction and anatomical localization. Two experienced nuclear medicine physicians who were blinded to the findings of clinical and prognostic information have reported ^18^F-FDG-PET/CT images by reaching consensus. The SUVmax generated from each patient was used in the final analysis.

### 2.3. Statistical Analysis

Statistical analysis was performed using IBM SPSS Statistics (IBM Corp., Released 2019, IBM SPSS Statistics for Windows, Version 26.0., Armonk, NY) and MedCalc Statistical Software version 18.2.1 (MedCalc Software bvba, Ostend, Belgium; http://www.medcalc.org; 2018). The measurement data with normal distribution were expressed as means and standard deviation and compared by independent sample's *t*-test. Data with non-normal distribution were presented in median and interquartile range (IQR), and the differences in median SUVmax across groups were evaluated using the Mann–Whitney *U* test for 2 groups or the Kruskal–Wallis test for more than 2 groups as appropriate. Two-sided *p* values of <0.05 were considered significant. Categorical variables were compared using the Pearson chi‐square test or Fisher's exact test (when there are one or some cells with expected frequencies less than 5). Using the statistically significant data obtained from the above parameters, the receiver operating characteristic (ROC) curves were generated and areas under the curves (AUCs) were calculated. The cutoff SUVmax for differentiation was determined based on the maximum Youden index. The corresponding sensitivity and specificity were calculated.

## 3. Results

### 3.1. Demographic and Clinical Features

**Figure 1:**
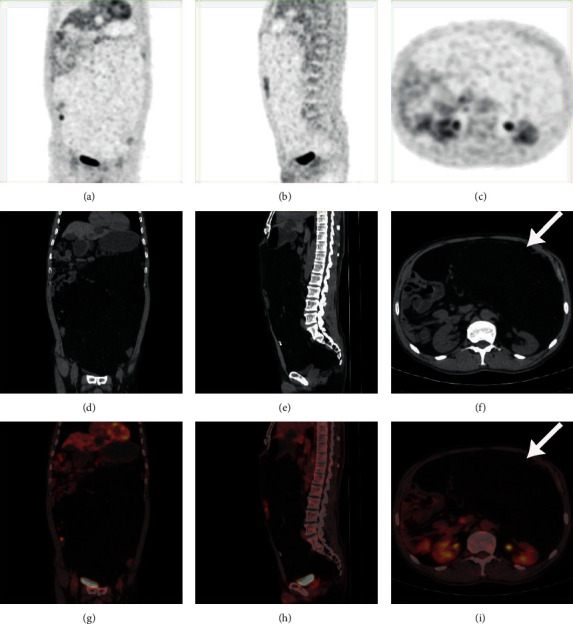
PET/CT images of a WDLPS patient with an SUVmax of 1.4 (white arrow).

**Figure 2:**
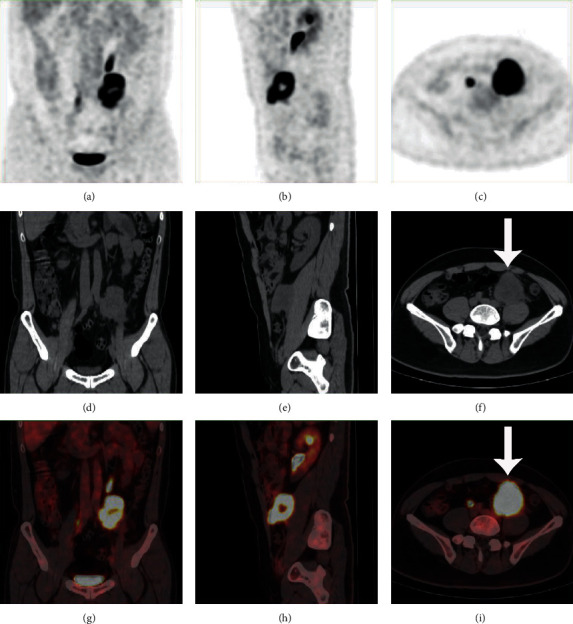
PET/CT images of a DDLPS patient with an SUVmax of 32.5 (white arrow).

A total of 84 RPLS patients (women = 37; men = 47) who underwent surgical resection from November 2013 to December 2019 at the sarcoma center of our institute were enrolled in this study. All patients did not accept any antitumor therapy before undergoing the PET/CT scan. Complete resection was achieved in 69 patients (82.1%), and incomplete resection was performed in 15 patients (17.9%). All patients received concomitant resection of a median of five additional organs (range: 1–10). The clinicopathological features of 84 patients are shown in Table 1. The median interval between PET/CT scan and surgery was 8 (IQR: 6–14.5) days. Forty-six patients (56.0%) had primary RPLS, and the remaining 37 patients (44.0%) had local recurrent RPLS. Regarding the histology, the predominant subtype was DDLPS in 60 patients (71.4%), WDLPS in 19 (22.6%), and PMLPS in 5 (6.0%). There were no statistically significant differences in the proportion of different histological subtypes or grades between primary and recurrent group (Table 2). The SUVmax of the entire cohort ranged from 1.4 to 32.5, and the median SUVmax was 5.2 (IQR: 3.0–9.2). The PET/CT images of a WDLPS patient (SUVmax = 1.4) and a DDLPS patient (SUVmax = 32.5) are demonstrated in Figures 1 and 2 separately. The median SUVmax was 4.8 (IQR: 2.9–9.3) and 6.0 (IQR: 3.2–9.0) in the primary and recurrent groups, respectively, and there was no statistically significant difference (*p* = 0.559) between the two groups.

### 3.2. Diagnostic Value for Predicting the Subtype

**Figure 3:**
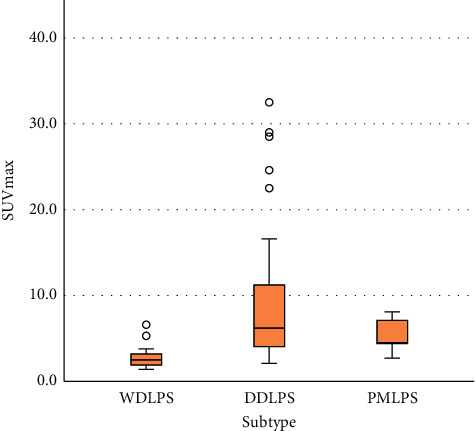
Distribution of SUVmax by histologic subtype.

**Figure 4:**
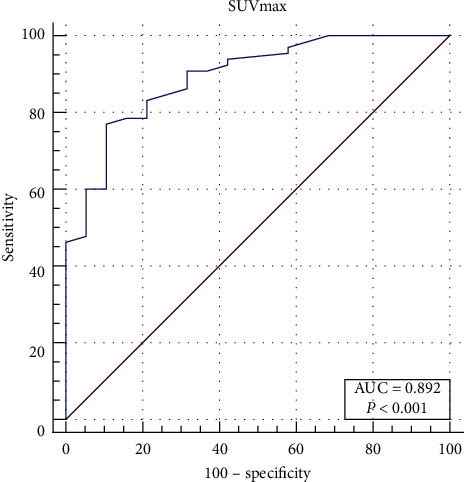
ROC curve for SUVmax for distinguishing WDLPS and non-WDLPS.

In the WDLPS group, the median SUVmax was 2.8 (IQR: 1.9–3.2), while those in the DDLPS and PMLPS were 6.2 (IQR: 4.1–11.3) and 4.5 (IQR4.0–7.4), respectively, showing a statistically significant difference (*p* < 0.001) between the median SUVmax across the three subtypes. The box plot of SUVmax for each pathological subtype is shown in Figure 3. By pairwise comparison of each subtype, the median SUVmax was statistically different between the WDLPS group and the other two groups (WDLPS-DDLPS: *p* < 0.001; WDLPS-PMLPS: *p* = 0.040) but showed no statistically significant differences between DDLPS and PMLPS groups (*p* = 0.459). The median SUVmax was statistically different between the WDLPS and non-WDLPS (DDLPS and PMLPS) groups (*p* < 0.001). The ROC curve suggested 3.8 as an appropriate cutoff value of SUVmax for distinguishing WDLPS and non-WDLPS (sensitivity = 0.769; specificity = 0.895, Figure 4). The AUC was calculated to be 0.892 (confidence interval (CI): 0.806–0.949, *p* < 0.001).

### 3.3. Diagnostic Value for Predicting FNCLCC Grade

**Figure 5:**
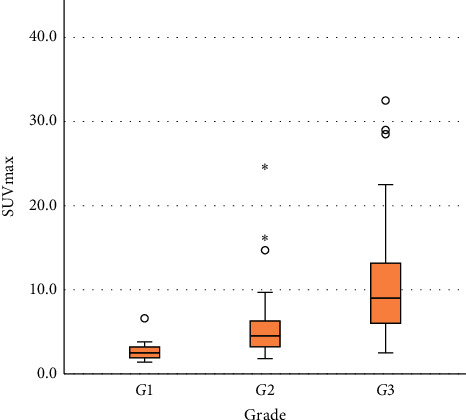
Distribution of SUVmax by FNCLCC grade.

**Figure 6:**
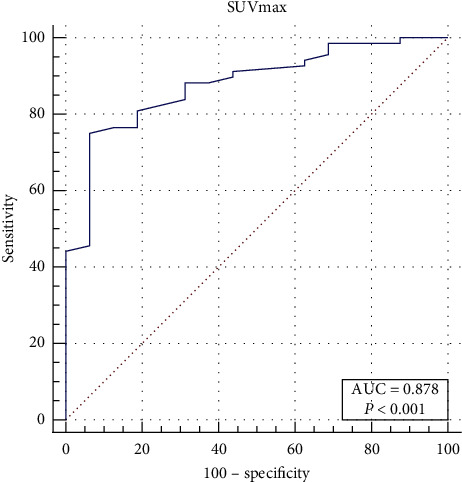
ROC curve for SUVmax for distinguishing FNCLCC G1 and G2-G3.

**Figure 7:**
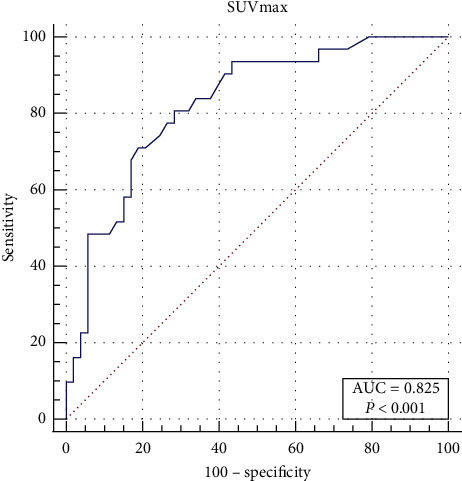
ROC curve for SUVmax for distinguishing FNCLCC G1-2 and G3.

The median SUVmax was 2.5 (IQR: 1.9–3.2), 4.5 (IQR: 3.2–6.7), and 9.0 (IQR: 6.0–13.3) for Grades 1, 2, and 3 groups separately, showing statistically significant differences among different grades. By pairwise comparison of each grade, the median SUVmax showed significant differences among two different grades (G1-G2: *p* = 0.002; G1-G3: *p* < 0.001; G2-G3: *p* = 0.001). The box plot of SUVmax for each tumor grade is shown in Figure 5. The ROC curve of SUVmax for predicting FNCLCC Grade 1 is shown in Figure 6. The AUC was calculated to be 0.878 (CI: 0.789–0.939, *p* < 0.001). By setting the cutoff point as SUVmax of 3.8, the sensitivity and specificity were calculated to be 75.0% and 93.8%, respectively. The ROC curve of SUVmax for predicting FNCLCC Grade 3 is shown in Figure 7. The AUC was calculated to be 0.825 (CI: 0.727–0.899, *p* < 0.001). By setting the cutoff point as SUVmax of 5.3, the sensitivity and specificity were calculated to be 80.7% and 71.7%, respectively.

## 4. Discussion

Accurate preoperative assessment of RPS has always been a critical issue. Contrast-enhanced CT scan is the preferred imaging study for diagnosis and staging of RPS. MRI on the other hand has superior contrast resolution and provides better tissue characterization [8]. Both CT and MRI are frequently used in clinical practice and, indeed, can provide detailed information with regard to tumor location, size, morphology, and structural changes. But neither of these can provide adequate information about tumor biology and malignant behavior. The combination of these two imaging technologies (^18^F-FDG PET/CT) provides information with regard to the location and metabolism of tumor. This in turn provides a whole-body imaging, detects the most aggressive portion of the tumor, and demonstrates the biological behavior of the tumor and therefore has a predictive value. These features are extremely important in detecting sarcomas that are commonly heterogeneous and pleomorphic.

In some reports, ^18^F-FDG PET/CT demonstrated high clinical efficacy for initial diagnosis, staging, guiding the appropriate site for biopsy, restaging, and predicting the prognosis and response assessment to therapy for the management of soft tissue sarcomas [9–14]. Additionally, previous studies have shown a significant correlation between SUVmax and tumor grading and have the ability in diffrentiating low-grade from high-grade sarcomas in suitable subtypes [15]. But most of these studies have limitations of inclusion of heterogeneous histological types and were mainly conducted on sarcomas that originated from the extremities and trunk. Only a few studies have been published on the role of ^18^F-FDG PET/CT in the diagnoses of RPS so far [4–6].

In this study, liposarcoma, which is the predominant histological type of RPS, was focused on. The results showed a high predictability of SUVmax for the subtype and FNCLCC grade. In general, the CT scan features of WDLPS or non-WDLPS are typical. Most of the WDLPS consisted of predominant fatty or large-area soft tissue density masses with uniform density and integrity margins containing minimal nodular nonlipomatous component and thin septa [8]. WDLPS usually has hypovascularity or slight enhancement, without visible calcification [16]. DDLPS is visualized as a predominant nonlipomatous mass, has frequent satellite nodules, and is sometimes presented as a dedifferentiated component within a WDLPS. About one quarter of DDLPS patients exhibited calcification or ossification within the tumor, which corresponded to metaplastic patterns of dedifferentiation [17, 18]. As an infrequent but highly malignant subtype, PMLPS had similar radiological features with that of DDLPS. PMLPSs often appear as large heterogeneous masses with areas of hemorrhage and necrosis and without any visible fat, making the imaging diagnosis difficult as they look like any other sarcoma [19]. Compared to DDLPS or PMLPS, cystic/necrotic areas were less commonly observed than in WDLPS [8, 16, 18]. Although the CT findings demonstrated different appearances between the subtypes, there were no unique characteristics observed.

As an effective diagnostic tool for evaluating both primary and locally or metastatic recurrent soft tissue sarcoma, the use of ^18^F-FDG PET/CT increases the accuracy in diagnosing different histological types and tumor grades of RPS. In this study, the result suggested SUVmax ≤3.8 as an appropriate cutoff value for diagnosing WDLPS and FNCLCC G1. Our study confirmed the results by MD Anderson cancer center, which included 14 DDLPS and 12 WDLPS with an SUVmax of 4.0 as a proper cutoff (area under the curve (AUC) = 0.875) for distinguishing WDLPS versus DDLPS [5]. Notably, the sample size of our study is quite larger than that of the MD Anderson series. In addition, our study also suggested an SUVmax of >5.3 as a proper cutoff value for predicting FNCLCC Grade 3 of RPLS. This result is similar to that of the previous Korean report that used SUVmax >4.5 as a cutoff point (AUC = 0.877) for diagnosing FNCLCC Grade 3 [6].

Although ^18^F-FDG PET/CT scan alone is not competent in diagnosing RPLS without contrast-enhanced CT or MRI, if a retroperitoneal tumor is suspected to be RLPS in CT or MRI scan, then ^18^F-FDG PET/CT scan can add valuable information with regard to the aggressiveness of the tumor. It is noteworthy that percutaneous biopsy has relatively low accuracy (36.5%) in diagnosing retroperitoneal DDLPS according to the previous report [20]. Accurate biopsy and adequate representative sampling in huge masses with heterogeneous components are sometimes difficult by conventional imaging modalities such as CT and MRI [21]. DDLPS is often presented as a dedifferentiated component within a WDLPS. If a sample is obtained only from the well-differentiated component of the mass, then the histologic diagnosis will probably be WDLPS. Our data suggest that ^18^F-FDG PET/CT for large heterogeneous RPLS provides guidance with regard to the presence of dedifferentiation and helps select most of the suitable biopsy sites. This study also supported the performance of ^18^F-FDG PET/CT in the initial diagnostic strategy for RPLS patients.

However, there are some limitations in this study. Firstly, there is an obvious selection bias. All the investigated patients underwent resection with definitive postoperative histological diagnosis. Many of the unresectable RPLS patients who underwent PET/CT were excluded as they had distant metastases or multifocal intraabdominal spread at the time of initial diagnosis. As a result, this study might not reflect the characteristics of the entire RPLS group. The unresectable RPLS patients with definitive histologic diagnosis should be enrolled to determine a more reliable result. Secondly, majority of the cases (94%) in this study are WDLPS and DDLPS. As an infrequent subtype, only five cases with PMLPS were enrolled in this study, and limited sample size is considered as a major confounding factor. Thirdly, 82.1% of the patients underwent complete resection and 17.9% of the patients underwent incomplete resection. In the latter group, accurate pathological diagnosis of some part of the samples might be missed, but the SUV index was measured for the whole tumor. Therefore, a potential mismatch is inevitable. Fourthly, only SUVmax was used to predict the subtype and tumor grade of RPLS. However, some parameters such as metabolic tumor volume (MTV) and total lesion glycolysis (TLG) have been reported to be useful for predicting the proliferative potential and prognosis of soft tissue sarcoma [22, 23]. Therefore, this study should be considered as a preliminary finding, and further studies are warranted to investigate the predictive value of MTV and TLG with regard to RPLS assessment.

In conclusion, this study is the largest Chinese monoinstitutional case-series of RPLS that presented high sensitivity and specificity by PET/CT for identifying the subtypes of RPLS, with an SUVmax cutoff of 3.8 to help differentiate between WDLPS and more aggressive histological types (DDLPS and PMLPS). Our study also suggested that SUVmax ≤3.8 and >5.3 can be used to predict FNCLCC Grades 1 and 3 of RPLS separately. These findings suggest that ^18^F-FDG PET/CT might be a useful complementary imaging modality for predicting the histologic subtype and tumor grade and guide suitable biopsy location in of RPLS. Based on the SUVmax of retroperitoneal sarcoma that is suspected to be liposarcoma, individualized surgical plans should be developed preoperatively according to the possible histological subtype.

## Figures and Tables

**Table 1:** Characteristics of 84 RPLS patients in the study.

Characteristics	No.	%
Age (years)		
Median (range)	57 (29–81)	
Sex		
Male	47	56.0
Female	37	44.0
Presentation		
Primary	46	54.8
Recurrent	38	45.2
Tumor size (cm)		
Median (range)	23 (4.6–50)	
Subtype		
WDLPS	19	22.6
DDLPS	60	71.4
PMLPS	5	6.0
FNCLCC grade		
G1	16	19.0
G2	37	44.0
G3	31	37.0

**Table 2:** Distributions of subtypes and grades of primary and recurrent RPLS.

	Primary	Recurrent	*p* value
Subtype			
WDLPS	14 (30.4%)	5 (13.2%)	*p* = 0.160
DDLPS	30 (65.2%)	30 (78.9%)	
MPLS	2 (4.3%)	3 (7.9%)	
FNCLCC grade			
G1	11 (23.9%)	5 (13.2%)	*p* = 0.458
G2	19 (41.3%)	18 (47.4%)	
G3	16 (34.8%)	15 (39.5%)	
Total	46 (100%)	38 (100%)	

## Data Availability

The data used to support the findings of this study are available from the corresponding author upon request.

## References

[1] Yang Z., Zheng R., Zhang S., Zeng H., Li H., Chen W. (2019). Incidence, distribution of histological subtypes and primary sites of soft tissue sarcoma in China. *Cancer Biology & Medicine*.

[2] Gronchi A., Miceli R., Allard M. A. (2015). Personalizing the approach to retroperitoneal soft tissue sarcoma: histology-specific patterns of failure and postrelapse outcome after primary extended resection. *Annals of Surgical Oncology*.

[3] Tan M. C. B., Brennan M. F., Kuk D. (2016). Histology-based classification predicts pattern of recurrence and improves risk stratification in primary retroperitoneal sarcoma. *Annals of Surgery*.

[4] Liu D. N., Li Z. W., Wang H. Y., Zhao M., Zhao W., Hao C. Y. (2018). Use of 18F-FDG-PET/CT for retroperitoneal/intra-abdominal soft tissue sarcomas. *Contrast Media Mol Imaging*.

[5] Parkes A., Urquiola E., Bhosale P. (2020). PET/CT imaging as a diagnostic tool in distinguishing well-differentiated versus dedifferentiated liposarcoma. *Sarcoma*.

[6] Rhu J., Hyun S. H., Lee K. H. (2019). Maximum standardized uptake value on 18 F-fluorodeoxyglucose positron emission tomography/computed tomography improves outcome prediction in retroperitoneal liposarcoma. *Science Reports*.

[7] Coindre J. M. (2006). Grading of soft tissue sarcomas: review and update. *Archives of Pathology & Laboratory Medicine*.

[8] Bajaj G., Tirumani H., Whisman M. K. (2020). Comprehensive review of abdominopelvic mesenchymal tumors with radiologic pathologic correlation and update on current treatment guidelines-Part 1. *Seminars in Ultrasound, CT and MRI*.

[9] Fuglø H. M., Jørgensen S. M., Loft A., Hovgaard D., Petersen M. M. (2012). The diagnostic and prognostic value of 18F-FDG PET/CT in the initial assessment of high-grade bone and soft tissue sarcoma. A retrospective study of 89 patients. *Diagnostic and Interventional Imaging*.

[10] Kassem T. W., Abdelaziz O., Emad-Eldin S. (2017). Diagnostic value of 18F-FDG-PET/CT for the follow-up and restaging of soft tissue sarcomas in adults. *Diagnostic and Interventional Imaging*.

[11] Kubo T., Furuta T., Johan M. P., Ochi M. (2016). Prognostic significance of 18F-FDG PET at diagnosis in patients with soft tissue sarcoma and bone sarcoma; Systematic review and meta-analysis. *European Journal of Cancer*.

[12] Li Y.-J., Dai Y.-L., Cheng Y.-S., Zhang W.-B., Tu C.-Q. (2016). Positron emission tomography (18)F-fluorodeoxyglucose uptake and prognosis in patients with bone and soft tissue sarcoma: a meta-analysis. *European Journal of Surgical Oncology (EJSO)*.

[13] Lim H. J., Johnny Ong C.-A., Tan J. W.-S., Ching Teo M. C. (2019). Utility of positron emission tomography/computed tomography (PET/CT) imaging in the evaluation of sarcomas: a systematic review. *Critical Reviews in Oncology/Hematology*.

[14] Shin D.-S., Shon O.-J., Han D.-S., Choi J.-H., Chun K.-A., Cho I.-H. (2008). The clinical efficacy of 18F-FDG-PET/CT in benign and malignant musculoskeletal tumors. *Annals of Nuclear Medicine*.

[15] Etchebehere E. C., Hobbs B. P., Milton D. R. (2016). Assessing the role of 18F-FDG PET and 18F-FDG PET/CT in the diagnosis of soft tissue musculoskeletal malignancies: a systematic review and meta-analysis. *European Journal of Nuclear Medicine and Molecular Imaging*.

[16] Lu J., Qin Q., Zhan L.-L. (2014). Computed tomography manifestations of histologic subtypes of retroperitoneal liposarcoma. *Asian Pacific Journal of Cancer Prevention*.

[17] Tateishi U., Hasegawa T., Beppu Y., Satake M., Moriyama N. (2003). Primary dedifferentiated liposarcoma of the retroperitoneum. *Journal of Computer Assisted Tomography*.

[18] Lahat G., Madewell J. E., Anaya D. A. (2009). Computed tomography scan-driven selection of treatment for retroperitoneal liposarcoma histologic subtypes. *Cancer*.

[19] Kim T., Murakami T., Oi H. (1996). CT and MR imaging of abdominal liposarcoma. *American Journal of Roentgenology*.

[20] Ikoma N., Torres K. E., Somaiah N. (2015). Accuracy of preoperative percutaneous biopsy for the diagnosis of retroperitoneal liposarcoma subtypes. *Annals of Surgical Oncology*.

[21] Park J. H., Park E. K., Kang C. H., Kim C. H., Choe J. G., Noh W. (2009). Intense accumulation of 18F-FDG, not enhancement on MRI, helps to guide the surgical biopsy accurately in soft tissue tumors. *Annals of Nuclear Medicine*.

[22] Choi E.-S., Ha S.-G., Kim H.-S., Ha J. H., Paeng J. C., Han I. (2013). Total lesion glycolysis by 18F-FDG PET/CT is a reliable predictor of prognosis in soft-tissue sarcoma. *European Journal of Nuclear Medicine and Molecular Imaging*.

[23] Kitao T., Shiga T., Hirata K. (2019). Volume-based parameters on FDG PET may predict the proliferative potential of soft-tissue sarcomas. *Annals of Nuclear Medicine*.

